# Detection of the Lunar Surface Soil Permittivity with Megahertz Electromagnetic Wave

**DOI:** 10.3390/s21072466

**Published:** 2021-04-02

**Authors:** Qingwen Rao, Guanjun Xu, Wangchen Mao

**Affiliations:** 1 Shanghai Key Laboratory of Multidimensional Information Processing, East China Normal University, Shanghai 200241, China; 51191214004@stu.ecnu.edu.cn; 2 Peng Cheng Laboratory, Shenzhen 518052, China; 3 Engineering Center of SHMEC for Space Information and GNSS, East China Normal University, Shanghai 200241, China; 4 School of Communication and Electronic Engineering, East China Normal University, Shanghai 200241, China; 10172100229@stu.ecnu.edu.cn

**Keywords:** lunar exploration, dielectric constant of lunar soil, dust plasma in the lunar ionosphere, improved scattering matrix method, electromagnetic wave

## Abstract

In this paper, the detection of the lunar surface soil permittivity with megahertz electromagnetic (EM) waves by spaceborne radar is studied based on the EM scattering theory, the Boltzmann–Shukla equations, and the improved scattering matrix method (ISMM). The reflection characteristics of the lunar surface soil subject to megahertz waves are analyzed through the EM scattering theory and expressed by the lunar surface soil permittivity. Then, the lunar ionosphere is assumed to be composed of dusty plasma, and its EM characteristics are described with the Boltzmann–Shukla equations. Finally, the transmission and reflection characteristics of the propagation of EM waves in the lunar ionosphere are numerically calculated with ISMM. Thus, the complex permittivity of lunar surface soil is obtained. In addition, the effects of detection environment situations, such as the lunar illumination intensity, characteristics of the lunar dust and dust charging process in the lunar ionosphere, on the amplitude and phase of EM waves are also investigated in this study. The simulation results show that an EM wave at a high frequency induces a strong effective wave with a stable phase shift and a significantly small interferential wave. Moreover, the lunar illumination is more effective under EM waves in low frequency bands; the characteristics of the lunar dust have a notable influence on the transmission and absorption coefficients of the effective waves. These conclusions help in real applications involving the detection of the lunar surface soil permittivity by spaceborne radar in various lunar environments.

## 1. Introduction

The Moon is the only satellite of the Earth, and the study of the Moon has great significance. In particular, it is imperative to detect the related parameters of the lunar surface topography and lunar soil medium. These together present the geological structure of the Moon, which can provide the basis for future detector landings [1]. At present, researchers have carried plenty of studies by using a radar to detect the dielectric constant of lunar soil. Yushkova and Yuhkov discussed the possibility of measuring the dielectric properties of lunar soil by using the bistatic radar method. Some methods to calculate the real part of the dielectric permittivity and the loss tangent of the lunar soil are proposed [2]. Hongo et al. further estimated the dielectric constant, porosity, and tangent of loss angle near the moon surface by the observed LRS data [3]. In addition, owing to the interaction between the solar wind and the lunar surface, the lunar surface is a very complex and changeable environment, which will cause obstacles for lunar exploration [4]. One of the most significant issues is that signals emitted from the probe will be attenuated, refracted, and reflected in the dusty plasma on the lunar surface, with the result that the probe will be unable to receive effective signals containing information about the lunar surface [5,6]. Therefore, the dusty plasma on the lunar surface poses a severe threat to the safety of landers and astronauts. Thus, there is a pressing need to study the propagation characteristics of electromagnetic (EM) waves in the dusty plasma on the lunar surface.

The dusty plasma on the lunar surface is composed of electrons, ions, charged dust particles, and neutral particles [7]. Therefore, the interaction between the EM waves and the dusty plasma is strongly related to the concentration and distribution of particles in the dusty plasma. The environment of the lunar surface has been well studied recently [8,9,10]. Based on the results of radio occultation measurements from the Soviet Lunar 19 mission, the research by Stubbs et al. suggests that the measurements from this probe were caused by the electrons emitted from exospheric dust and this process could be responsible for the formation and evolution of the lunar ionosphere [11]. In addition, Thompson et al. described the surface soil as a non-dispersive medium, which enables convenient radar detection on the lunar surface soil [12].

Based on the previous investigations on the lunar surface environment, plenty of works on lunar communication have been carried out. Hwu et al. analyzed the effects of the lunar surface environment on wireless communication [13]. Their results indicate that path loss in the lunar surface environment is much more severe than free-space propagation and is affected by the operating frequency and lunar surface soil. In addition, the study by Pabari et al. showed that deploying a relaying sensor is an alternative way of ensuring lunar communication under the area coverage with a constrained signal strength [14]. Some meaningful works focusing on the interaction between EM waves and the dusty plasma have been conducted. Baishya and Das studied the dynamics of dust particles in a magnetized dusty plasma [15]. Cao et al. analyzed the propagation characteristics of an EM wave in a non-uniform dusty plasma [16]. Moreover, Chen et al. studied the effects of a fully ionized dusty plasma on the wave propagation [17]. According to the above review, some potential schemes have been proposed for lunar communication, and the influence of the dusty plasma on the communication system has been investigated in previous studies. However, the propagation properties of the EM in the lunar dusty plasma, which comes ultimately from the lunar ionosphere and could reflect the detection signal, resulting in the interruption of communication, have rarely been studied, to the best of our knowledge.

In this paper, an algorithm for calculating the dielectric constant of lunar surface soil is proposed, and the influence of interference signals is also considered in this method. Moreover, the influence of the dusty plasma on the characteristics of EM waves is simulated and analyzed within the improved scattering matrix method (ISMM) [18,19]. The effects of electron density, dust particles, and collision frequency on the transmission, reflection, and phase are discussed.

The rest of this paper is organized as follows. In Section 2, the physical model and environment parameters are introduced. In addition, the transmission and reflection of the ionosphere are derived. The reflection of the lunar surface soil is also calculated by the complex permittivity of the lunar surface soil. In Section 3, the effect of the lunar environment, such as the lunar illumination intensity, the characteristics of the dust, and the dust charging process, are shown and analyzed. Conclusions are finally summarized in Section 4.

## 2. Physical Model and Formulations

### 2.1. Physical Model and Lunar Ionosphere

**Figure 1:**
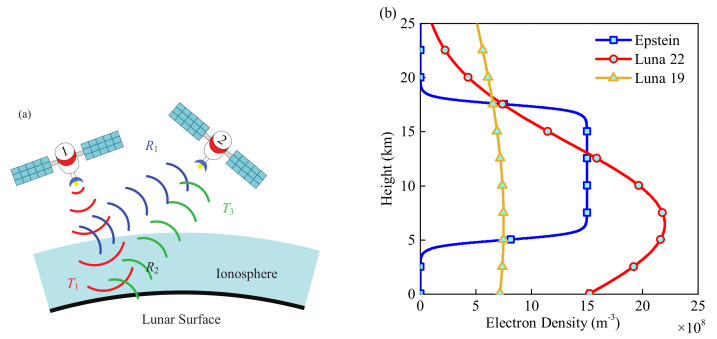
(**a**) Schematic of spaceborne radar detection and (**b**) the electron density profile of lunar ionosphere.

In this paper, radar detection is divided into three steps, shown with different colors in Figure 1a. A satellite emits an EM wave to the lunar surface, which is marked with red color. When the EM wave passes through the lunar ionosphere, a part of the EM wave will be reflected by the lunar ionosphere, which is marked with blue color. The ratio of the reflected EM wave to the incident wave is denoted by $R_{1}$, and the ratio of the transmitted EM wave is denoted by $T_{1}$. The transmitted EM wave will be reflected at the lunar surface soil by the coefficient of $R_{2}$, which contains the information of the lunar surface soil permittivity. The reflected wave passes back through the lunar ionosphere in reverse and the transmission coefficient is denoted by $T_{3}$. It is worth noting that $T_{1}$ is different from $T_{3}$. Therefore, a satellite can capture two kinds of reflected waves: wave carrying information about the lunar surface soil permittivity, known as effective wave, and wave totally caused by the reflection of the ionosphere, known as interferential wave. In addition, the coefficient the effective waves and interferential waves are denoted $R_{Eff}$ and $R_{Int}$, respectively, and $R_{Eff}$ and $R_{Int}$ can be theoretically calculated as

(1) $$R_{Eff}=T_{1}\times R_{2}\times T_{3}.$$

(2) $$R_{Int}=R_{1}.$$

Above, $T_{1}$, $T_{3}$ and $R_{1}$ can be obtained by the simulation between the lunar ionosphere and the EM wave. With the practically captured $R_{Eff}$, the reflection coefficient of the lunar surface soil, $R_{2}$, can be figured out. Furthermore, $R_{2}$ can be used to calculate the lunar surface soil permittivity, which will be shown in Section 2.2.

Figure 1b shows three electron density profiles of the lunar ionosphere versus the height from the lunar surface. In Grobman’s study, an ionosphere with nearly even distribution is theoretically derived at the height of 5–20 km [20]. Therefore, an electron density profile in Epstein’s distribution is employed in this study for simulation:

(3) $$N_{e}=\left\{\begin{array}{c} \frac{N_{0}}{1+\exp\left(\frac{L-4h}{4\sigma }\right)}\\ \frac{N_{0}}{1+\exp\left(\frac{h-3L}{4\sigma }\right)}\\ 0 \end{array}\begin{array}{c} ,\left(0<h<\frac{2L}{5}\right)\\ ,\left(\frac{2L}{5}<h<\frac{4L}{5}\right)\\ ,\left(\frac{4L}{5}<h<L\right) \end{array}\right..$$

Here, the total thickness of the lunar ionosphere, *L*, is set as $2.5\times 10^{4}$ m, and the maximum of the electron density is denoted by $N_{0}$, set as $1.5\times 10^{9}$ $m^{-3}$ [21]. The gradient scale factor in Epstein’s distribution is set as $\sigma =3\times 10^{4}$. The other two density profiles are fitting curves in the Maxwell–Boltzmann distribution from actual data collected by Luna 22 & 19 [9,10]. The total electron content (TEC) of the three electron distribution profiles is $2.59\times 10^{13}$ $m^{-2}$, $3.31\times 10^{13}$ $m^{-2}$, and $1.70\times 10^{13}$ $m^{-2}$, respectively.

### 2.2. The Permittivity and Reflection of the Lunar Surface Soil

**Figure 2:**
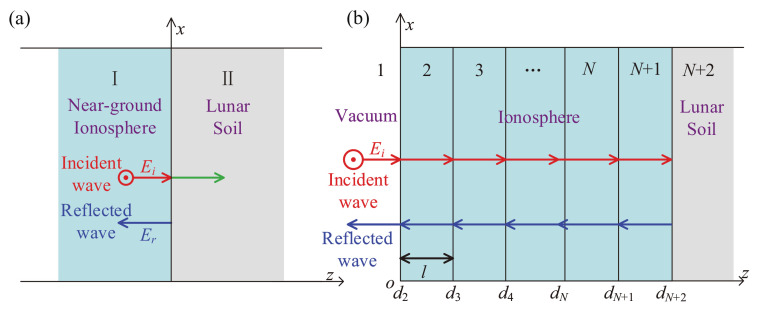
Schematic of lunar surface soil reflection and waves propagation in the lunar ionosphere. (**a**) the local enlarged schematic diagram of the N+1 and N+2 layers in (**b**), (**b**) the schematic diagram of electromagnetic wave propagation in the stratified dusty plasma.

The transmitted EM wave is reflected by the lunar surface soil, as shown in Figure 2a. At the interface between the near-ground ionosphere and the lunar surface soil, the electric field of the incidence EM wave is set as

(4) $$\begin{array}{c} \vec{E_{i}}=\vec{e_{y}}E_{0}\exp\left[-j\left(k_{1}z-\omega t\right)\right], \end{array}$$

where $\vec{e_{y}}$ is the unit vector of the electric field along the *y*-axis, $E_{0}$ is the amplitude of the electric field, $k_{1}$ is the propagation constant in vacuum, ω is the angular frequency of EM waves, *z* is the distance of EM wave propagate in *z*-axis, and *j* is an imaginary unit.

Considering the consistency of the tangential components for the electric fields at the two sides of the interface, the electric field of the reflected EM wave can be expressed as

(5) $$\begin{array}{c} \vec{E_{r}}=\vec{e_{y}}\left(R_{2}\times E_{0}\right)\exp\left[-j\left(-k_{1}z-\omega t\right)\right], \end{array}$$

Equation (5) represents the relationship between the reflected wave and the incident wave on the boundary, and $R_{2}$ can be calculated as

(6) $$R_{2}=\frac{\sqrt{\varepsilon_{r,\mathrm{II}}}-\sqrt{\varepsilon_{r,I}}}{\sqrt{\varepsilon_{r,\mathrm{II}}}+\sqrt{\varepsilon_{r,I}}},$$

with $\varepsilon_{r,I}$ means the complex permittivity of the near-ground ionosphere. In the study by Thompson, the lunar surface soil is described as a non-dispersive medium [12]. Therefore, the complex permittivity of the lunar surface soil, $\varepsilon_{r,\mathrm{II}}$, can be expressed as

(7) $$\varepsilon_{r,\mathrm{II}}={\varepsilon_{\mathrm{II}}}^{\prime }-j\times {\varepsilon_{\mathrm{II}}}^{\prime }\times \tan\delta_{\varepsilon },$$

where ${\varepsilon_{\mathrm{II}}}^{\prime }$ is the real part of the complex permittivity and $\tan\delta_{\varepsilon }$ is the dielectric loss angle.

### 2.3. Dusty Plasma Permittivity and ISMM

In the Apollo project, the lunar ionosphere was detected to be composed of plasma with much active dust that differs from normal plasma in its charging properties [22]. Considering the Boltzmann–Shukla equations, the relative complex permittivity of the weakly ionized dusty plasma can be calculated from [23,24]

(8) $$\begin{array}{c} \varepsilon_{r}=1-\frac{\omega_{p}^{2}}{\omega^{2}+\nu_{en}^{2}}+\frac{c\eta_{ed}\left(\nu_{ch}+\nu_{en}\right)}{\varepsilon_{0}\left(\omega^{2}+\nu_{en}^{2}\right)\left(\omega^{2}+\nu_{ch}^{2}\right)}+j\frac{1}{\varepsilon_{0}\omega }\left[\frac{\varepsilon_{0}\omega_{p}^{2}\nu_{en}}{\omega^{2}+\nu_{en}^{2}}+\frac{c\eta_{ed}\left(\omega^{2}-\nu_{ch}\nu_{en}\right)}{\left(\omega^{2}+\nu_{en}^{2}\right)\left(\omega^{2}+\nu_{ch}^{2}\right)}\right]. \end{array}$$

Here, $\varepsilon_{0}$ and *c* are constants corresponding to the vacuum permittivity and the speed of light in free space. ω is the angular frequency of the incidence wave. The plasma frequency, $\omega_{p}$, can be calculated from the plasma electron density, $N_{e}$, by $\omega_{p}=e\sqrt{N_{e}/\left(m_{e}\varepsilon_{0}\right)}$, where $m_{e}$ represents the single electron mass. $\eta_{ed}$ denotes the charging response factor of the dust, which can be obtained as [25,26]

(9) $$\eta_{ed}=e^{2}\pi r_{d}^{2}N_{e}N_{d}/m_{e},$$

where $r_{d}$ is the radius of dust in the lunar ionosphere; $N_{d}$ represents the dust density. The dusty particle charging frequency, $v_{ch}$, and the plasma collisional frequency, $v_{en}$, are also important parameters to describe the dusty plasma. However, in the environment of the lunar ionosphere, $v_{en}$ is set as 0.

To calculate the transmission and reflection coefficient ($T_{1},R_{1},$ and $T_{3}$), the physical model of the lunar ionosphere in a stratified structure is established in Figure 2b. Some basic assumptions are set as follows to use the ISMM for simulation. Since the satellite is far from the lunar surface, the left side of the ionosphere in Figure 2b is assumed as a vacuum. As for stratification, the ionosphere is evenly divided into *N* sub-layers, and the thickness of each layer is set to be l=L/N. For the dusty plasma in each ionosphere layer, the electron density, charging response factor, and other parameters are all uniformly distributed.

A linearly polarized EM wave propagates from vacuum vertically into the lunar ionosphere layers, whose electric field is parallel to the *y*-axis and whose magnetic field is parallel to the *x*-axis. With the reference point at the left interface of each layer, $d_{m}$, the electric field of the EM wave in the *m*-th ionosphere layer can be expressed as

(10) $$\begin{array}{c} \vec{E_{m}}\left(z\right)=\vec{e_{y}}E_{0}\left\{A_{m}\exp\left\{-j\left\{k_{m}\left[z-\left(m-2\right)\times l\right]-\omega t\right\}\right\}\right.+\\ \left.B_{m}\exp\left\{-j\left\{-k_{m}\left[z-\left(m-2\right)\times l\right]-\omega t\right\}\right\}\right\} \end{array},$$

where $k_{m}$ means the wave number of the EM wave in the *m*-th ionosphere layer and can be calculated by $k_{m}=\omega /c\sqrt{\varepsilon_{r,m}}$. For clarity, the first term on the right side of Equation (10), $A_{m}$, represents the sum of the EM wave propagating along the positive direction of the *z*-axis in the ionosphere layers; the second term, $B_{m}$ represents the sum of the EM wave propagating along the negative direction of the *z*-axis. When m=1, in the leftmost vacuum, $A_{1}=1$ means the total incident EM wave; $B_{1}=r$ means the coefficient of the EM wave reflected into vacuum by the lunar ionosphere. When m=N+1, in the rightmost ionosphere layer, $A_{N+1}=t$ means the coefficient of transmission; $B_{N+1}=0$ means the absence of a return wave from vacuum.

With Faraday’s electromagnetic induction law in an ideal conductive medium,

(11) $$\nabla \times \vec{E}=-j\mu \omega \vec{H},$$

the total magnetic field in the *m*-th ionosphere layer can be obtained from Equation (10) as

(12) $$\begin{array}{c} \vec{H_{m}}\left(z\right)=\vec{e_{x}}\frac{k_{m}E_{0}}{\mu \omega }\left\{-A_{m}\exp\left\{-j\left\{k_{m}\left[z-\left(m-2\right)\times l\right]-\omega t\right\}\right\}\right.. \end{array}$$

Both Equations (10) and (12) are substituted into the boundary conditions and the results are further simplified into a matrix equation

(13) $$\left[\begin{array}{c} A_{m+1}\\ B_{m+1} \end{array}\right]=S_{m}\left[\begin{array}{c} A_{m}\\ B_{m} \end{array}\right],$$

where $S_{m}$ is the transfer matrix of the *m*-th ionosphere layer and is described as

(14) $$S_{m}={\left[\begin{array}{cc} 1 & 1\\ k_{m+1} & -k_{m+1} \end{array}\right]}^{-1}\left[\begin{array}{cc} \exp\left(-jk_{m}l\right) & \exp\left(jk_{m}l\right)\\ k_{m}\exp\left(-jk_{m}l\right) & -k_{m}\exp\left(jk_{m}l\right) \end{array}\right].$$

By iterating Equation (13) from the 2nd to the (*N*+1)-th layer, a total matrix equation is obtained as

(15) $$\left[\begin{array}{c} A_{N+1}\\ B_{N+1} \end{array}\right]=\prod_{m=1}^{N}S_{m}\left[\begin{array}{c} A_{1}\\ B_{1} \end{array}\right].$$

With $A_{1}=1$, $B_{1}=r$, $A_{N+1}=T$, $B_{N+1}=0$, and

(16) $$S_{g}=\left[\begin{array}{cc} S_{g11} & S_{g12}\\ S_{g21} & S_{g22} \end{array}\right]=\prod_{m=1}^{N}S_{m},$$

the total coefficient of the transmission and reflection of the EM wave can therefore be obtained as

(17) $$\left\{\begin{array}{c} T=S_{g11}-\frac{S_{g12}\times S_{g21}}{S_{g22}}\\ R=-\frac{S_{g21}}{S_{g22}} \end{array}\right..$$

With Equation (17), $T_{1}$, $T_{3}$ and $R_{1}$ in Equation (1) can be calculated. Notably, both the magnitude change and phase shift are reserved in the resulting complex coefficient of reflection or transmission. The advantage of the ISMM in analyzing the permittivity of the lunar surface soil from the captured $R_{Eff}$ will be discussed in Section 3.1.

## 3. Results and Discussion

As shown in Section 2, the ${\varepsilon_{\mathrm{II}}}^{\prime }$ and $\tan\delta_{\varepsilon }$ are calculated via Equation (1) and (6) and the interferential wave is estimated via Equation (2). Here, $P_{t}={\left|T_{1}\right|}^{2}{\left|T_{3}\right|}^{2}$ and $P_{r}=-\log\left({\left|R_{1}\right|}^{2}\right)$ (dB) are used for the sake of discussion and analysis.

### 3.1. Common Detection with Three Typical Missions

**Figure 3:**
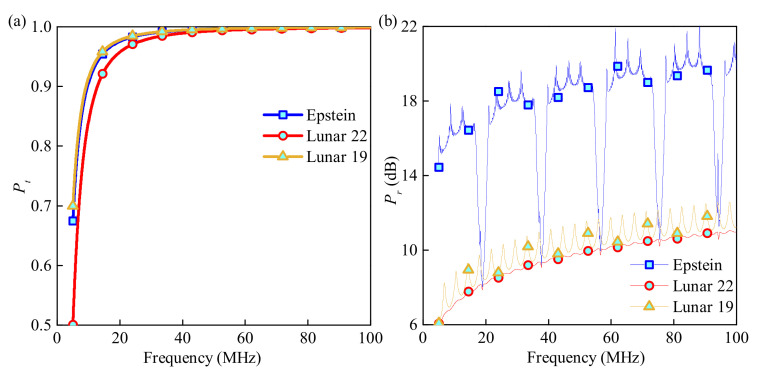
(**a**) $P_{t}$ and (**b**) $P_{r}$ versus wave frequency under three ionosphere profiles.

Both $P_{t}$ and $P_{r}$ versus wave frequency under three ionosphere profiles are depicted in Figure 3. Some key parameters are set as: the radius of dust particles, $r_{d}$, is $9\times 10^{-6}$ m; the dust density of the ionosphere, $N_{d}$, is $8\times 10^{7}$ $m^{-3}$; the dusty particle charging frequency, $v_{ch}$, is set as 0.3 GHz, the number of sublayers, *N*, is 500. From Figure 3a, for frequency bands over 20 MHz, $P_{t}$ for the three profiles are all over 90%; for frequency bands over 40 MHz, the EM wave can almost totally penetrate the lunar ionosphere. From Figure 3b, for the frequency band between 5–100 MHz, the $P_{r}$ is always higher than 6 dB, which is much smaller than the $P_{t}$. Therefore, the effective wave can be captured, distinguished, and excluded. Overall, an EM wave with a high frequency is easier to transmit forth and back in the lunar ionosphere and induces a small coefficient of interferential wave. In summary, Figure 3 demonstrates the feasibility of this radar detection method and shows the general law of the captured wave with the wave frequency by simulation.

### 3.2. Lunar Illumination Intensity

The lunar ionosphere is formed mainly because of photoionization. From the Saha formula [27], the more energy the photon from the Sun gives to the neutral particles in the lunar ionosphere, the greater is the degree of ionization and the more active electrons the ionosphere contains [28]. During the detection, the lunar ionosphere is inevitably affected by the environment conditions of day, night, or even the solar wind, which results in fluctuations of the TEC. Therefore, the following study is carried out on the effect of the lunar illumination intensity on the coefficient of the effective and interferential wave. However, due to the microgravity of the Moon, electrons with enormous energy have a great potential to escape from the lunar ionosphere, which means a relationship between the illumination intensity and the TEC. Therefore, the TEC is directly employed in the simulation to calculate the $P_{t}$ and $P_{r}$ under different lunar illumination intensities.

**Figure 4:**
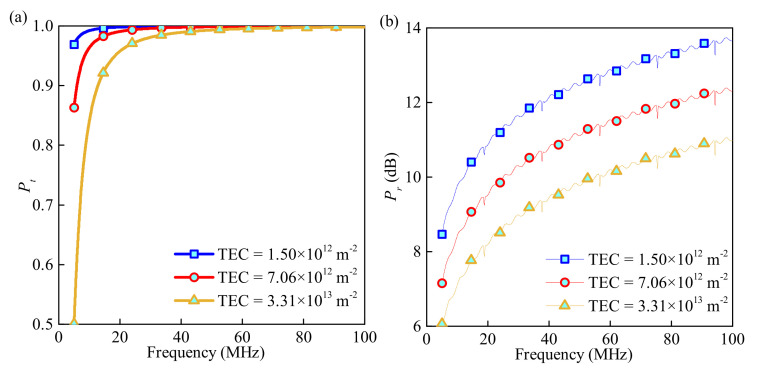
(**a**) $P_{t}$ and (**b**) $P_{r}$ versus wave frequency under three TECs.

Above, the electron profile from Luna 22 is employed and the TEC is set to be $1.5\times 10^{12}$ $m^{-2}$, $7.06\times 10^{12}$ $m^{-2}$, and $3.31\times 10^{13}$ $m^{-2}$ in turn. Other parameters are consistent with those in Figure 3. From Figure 4a, the attenuation in $P_{t}$ in a low frequency is more obvious than that of a high frequency. However, an EM wave over 40 MHz has a nearly complete transmission. The microgravity of the Moon creates an ionosphere with low TEC, which indicates less energy absorbed by the ionosphere and allows the high frequency wave to propagate. From Figure 4b, the TEC of the ionosphere has a slight impact on $P_{r}$. The $P_{r}$ is greater when the TEC is larger. However, the $P_{r}$ is small enough to be excluded overall. Therefore, a larger TEC induces a small $P_{t}$ and a slightly large $P_{r}$. The regulation of EM wave transmission and reflection in Figure 4 is not contradictory to results in other studies [29].

### 3.3. Characteristics of Dust Particles

**Figure 5:**
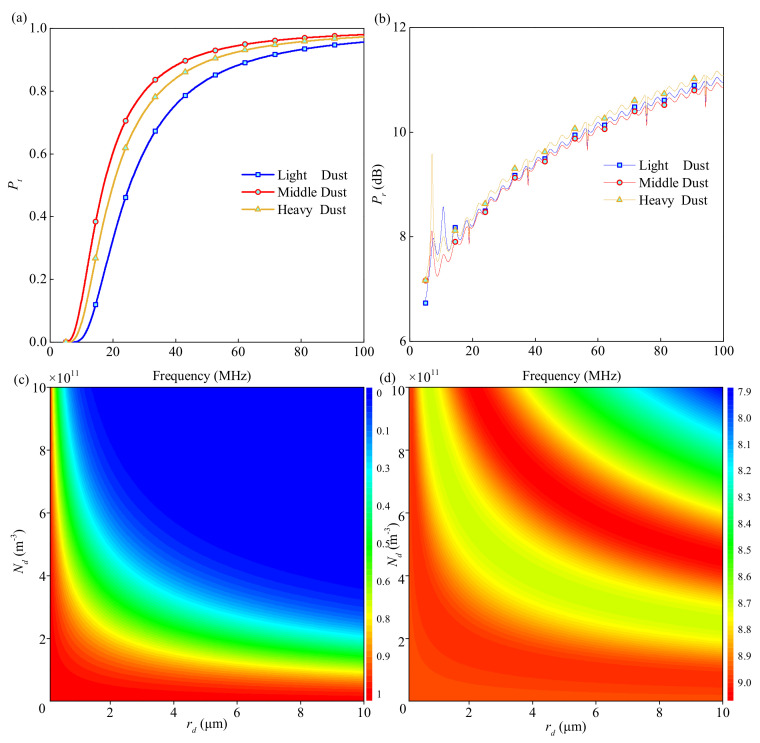
(**a**) $P_{t}$ and (**b**) $P_{r}$ versus wave frequency under three dust characteristics; (**c**) $P_{t}$ and (**d**) $P_{r}$ under dust characteristics of density and radius.

The lunar ionosphere is very close to the lunar surface, so a large amount of the lunar dust floats in the lunar ionosphere. Considering the difference of dust characteristics in different parts of the lunar, the dust in the lunar ionosphere is variable, particularly in its radius and density. To estimate the influence of variable dust characteristics, three representative dust particles are set as [30]: light dust, $r_{d}=4.58$ μm, $N_{d}=8\times 10^{9}$ $m^{-3}$; medium dust: $r_{d}=8.71$ μm, $N_{d}=1\times 10^{9}$ $m^{-3}$, and heavy dust: $r_{d}=18.66$ μm, $N_{d}=3\times 10^{8}$ $m^{-3}$. In addition, $P_{t}$ and $P_{r}$ are shown in Figure 5a,b with the increase of the wave frequency from 5 to 100 MHz.

From Figure 5a, the dust characteristics have a greater influence on $P_{t}$ than that of the TEC. Among these three dust conditions, light dust absorbs the most wave energy, followed by heavy dust and medium dust in turn. From Figure 5b, the dust has nearly no effect on $P_{r}$. For more detail, Figure 5c,d show $P_{t}$ and $P_{r}$ in the $N_{d}$ and $r_{d}$ bounds of 0–10 μm and 0–10 $\times 10^{11}$ $m^{-3}$. From Figure 5c, a higher dust density and larger dust radius can lead to a smaller $P_{t}$, respectively. The figure also shows the co-determination of $P_{t}$ by $N_{d}$ and $r_{d}$ in all cases. From Figure 5d, a higher dust density and larger dust radius can lead to a stronger $P_{r}$ overall. However, a band with small $P_{r}$ exists between the middle $P_{r}$ bands, where the interferential wave is meant to be rather faint. In the same region in Figure 5c, the $P_{t}$ is quite small and this small $P_{r}$ band may not be used in real applications. These figures show the effect of the radius and the density on the reflection. In view of the complexity of the dust in the lunar ionosphere, enhancing the frequency may be the only method to systematically strengthen the $P_{t}$ to obtain more accurate radar detection.

### 3.4. Dust Particle Charging Process

**Figure 6:**
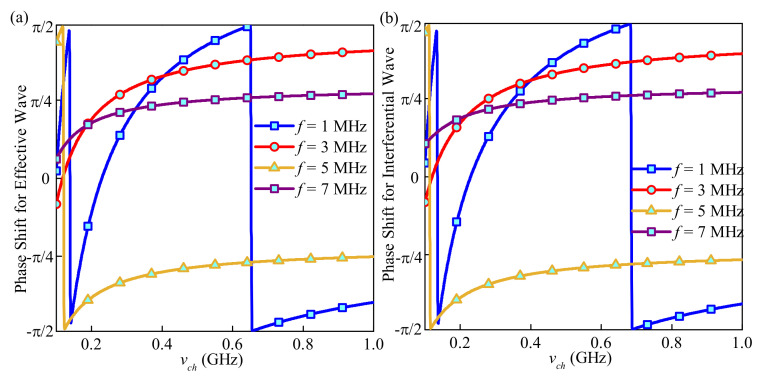
Phase shift for the (**a**) effective wave and (**b**) interferential wave versus dust charging frequency under four wave frequencies.

Dusty plasma differs from normal plasma in its charging process, and the charging process can affect the wave phase shift greatly, as is summarized in Vladimirov’s study [31]. The calculation of phase shift caused by ionosphere depends on the transmission coefficient calculated by ISMM, which describes the complex amplitude ratio of electromagnetic wave before and after penetrating the ionosphere. The ionosphere induced phase shift can be obtained by calculating the arctangent of the real part and imaginary part of the transmission coefficient. In the following simulation, the phase shift is shown and concluded from Figure 6.

From Figure 6, the phase shift for the effective and interferential wave is generally the same, which makes identification from the phase difference a problem. However, considering the very sparsity of the lunar ionosphere, that is, the very low electron concentration, we use a higher frequency of the probe wave, megahertz, which can explain the similar phase shift of the effective wave and the interferential wave. In addition, from another aspect, for the EM wave of 1 MHz, the phase shift jumped at about 0.14 and 0.65 GHz. The jumping frequency point increases trouble for the wave signal analysis. In addition, the system achieves a more stable phase when the wave frequency is over 7 MHz.

## 4. Conclusions

Based on the EM scattering theory, Boltzmann–Shukla equations, and the improved scattering matrix method, the complex permittivity from both the amplitude and phase shift of the reflection wave is investigated in this paper. In addition, different environment situations that will be encountered in the detection are introduced and simulated preliminarily. The simulation results show that detection in common situations captures large effective waves and small interferential waves, which indicates that it is a promising detection method. The influence of the lunar illumination intensity and the characteristics of dust in the lunar ionosphere are also estimated. A lower TEC induces a stronger effective wave and a faint interferential wave, while the effect of the dust particles is determined by both the radius and density together. Finally, the phase shift of the effective and interferential waves caused by the dust charging process is calculated. Our theoretical research provides a fundamental basis for the real application involving lunar spaceborne radar detection.

## Data Availability

Not applicable.
